# Impact of Worry on Experimental Hippocampal Tasks and Hippocampal Volumetrics in Older Adults with Subjective Cognitive Complaints

**DOI:** 10.1002/alz70857_103370

**Published:** 2025-12-25

**Authors:** Rachel N Schade, Andrew O'Shea, Katie Rodriguez, Joshua Gertler, Zhiguang Huo, Steven T. DeKosky, Adam J. Woods, Gene Alexander, Dawn Bowers

**Affiliations:** ^1^ University of Florida, Gainesville, FL, USA; ^2^ University Of Florida, Gainesville, FL, USA; ^3^ McKnight Brain Institute, University of Florida, Gainesville, FL, USA; ^4^ The University of Texas at Dallas, Dallas, TX, USA; ^5^ University of Arizona, Tucson, AZ, USA; ^6^ University of Florida Fixel Institute of Neurological Diseases, Gainsville, FL, USA

## Abstract

**Background:**

Subjective cognitive complaints (SCC) have been associated with decreased hippocampal volume and those with distress about their SCC have a higher risk of preclinical Alzheimer's disease (AD). The current study divided individuals with SCC into “worried” and “non‐worried” groups to determine whether 1) experimental hippocampal tasks and hippocampal volumetrics differ between groups and 2) determine if mood and/or motivation were driving differences.

**Method:**

Participants were 154 adults (mean age = 71±5 years, mean education = 16±2 years, 62% female, 87% white) with SCC. There were two groups: “worried” (*N* = 76) or “non‐worried” (*N* = 78) about cognitive changes over the past five years (Cognitive Change Index). Cognitive (two hippocampal‐based experimental tasks: ARENA (spatial navigation) and the Mnemonic Similarity Task (MST; pattern separation)), mood (Beck Depression Inventory‐II (BDI‐II), Apathy Scale (AS), and the State‐Trait Anxiety Inventory (STAI)), and neuroimaging data were drawn from the baseline visit. Hippocampal imaging was performed using high‐resolution T2 sequences (0.39x0.39mm). Whole hippocampal and CA1, CA3, and dentate gyrus volumetrics were analyzed with Freesurfer version 7.1.1. based on proposed neural correlates of ARENA and MST. Data were analyzed with independent t‐tests and ANCOVAs, with demographic and mood/motivation as covariates.

**Results:**

The worried group performed significantly worse on ARENA learning (*t*(152)=‐2.7, *p* = 0.004), recall (*t*(152)=‐2.2, *p* = 0.02), and overall composite memory z‐scores (*t*(152)=3.3, *p* < 0.001). There were no significant differences observed on the MST or hippocampal volumetrics between groups. The worried group reported greater depression (*t*(152)=‐2.4, *p* = 0.01), trait anxiety (*t*(152)=‐2.6, *p* = 0.01), apathy (*t*(152)=‐2.2, *p* = 0.01) on self‐report measures. ANCOVAs revealed that apathy significantly contributed to ARENA performance on learning (*F*(1,152)=6.7, *p* = 0.01) and recall composites (*F*(1,152)=5.4, *p* = 0.02), reducing group difference on learning composite only. Overall memory composite remained significant without demographic or mood contributions (*F*(1,152)=6.9, *p* = 0.009).

**Conclusions:**

Older adults with SCC worry exhibit higher levels of mood and motivation symptoms, which may contribute to poorer performance on cognitive tasks. Two hypotheses are possible: 1) greater mood symptoms are driving SCC and ARENA performance, or 2) ARENA may be uniquely sensitive to detect memory changes, as its neural correlates are implicated in early AD. Since mood symptoms are common in preclinical AD, the worried SCC group may reflect this preclinical change.

